# Impact of simulation-based teamwork training on COVID-19 distress in healthcare professionals

**DOI:** 10.1186/s12909-020-02427-4

**Published:** 2020-12-21

**Authors:** Anna Beneria, Mireia Arnedo, Sofia Contreras, Marco Pérez-Carrasco, Itziar Garcia-Ruiz, Mónica Rodríguez-Carballeira, Joaquim Raduà, Jordi Bañeras Rius

**Affiliations:** 1 Departament Psiquiatria, Vall d’Hebron Hospital Universitari, Barcelona, Spain; 2 Vall Hebron centre Simulació Clínica Avançada (VHiSCA), Direcció Docència, Vall d’Hebron Hospital Universitari, Vall d’Hebron Barcelona Hospital Campus, Barcelona, Spain; 3 Servei de Medicina Intensiva, Vall d’Hebron Hospital Universitari, Barcelona, Spain; 4 Unitat Medicina Materna i Fetal, Department d’Obstetrícia, Vall d’Hebron Hospital Universitari, Universitat Autònoma de Barcelona, Barcelona, Spain; 5 Institut d’Investigacions Biomèdiques August Pi i Sunyer (IDIBAPS), Barcelona, Spain; 6 CIBERSAM, ISC-III, Madrid, Spain; 7 King’s College London, London, UK; 8 Karolinska Institutet, Stockholm, Sweden; 9 Cardiology Vall d’Hebron Hospital Universitari, 08036 Barcelona, Spain

**Keywords:** COVID19 distress, Healthcare workers wellbeing, Simulation and teamwork

## Abstract

**Context:**

Non-technical skills such as leadership, communication, or situation awareness should lead to effective teamwork in a crisis. This study aimed to analyse the role of these skills in the emotional response of health professionals to the COVID-19 pandemic.

**Methods:**

Before the COVID-19 outbreak, 48 doctors and 48 nurses participated in a simulation-based teamwork training program based on teaching non-technical skills through simulation. In May 2020, this group of professionals from a COVID-19 referral hospital was invited to participate in a survey exploring stress, anxiety, and depression, using the PSS-14 (Perceived Stress Scale) and the HADS (Hospital Anxiety and Depression Scale) measures. A control group that did not receive the training was included. We conducted a logistic regression to assess whether having attended a simulation-based teamwork training program modified the probability of presenting psychological distress (PSS-14 > 18 or HADS> 12).

**Results:**

A total of 141 healthcare professionals were included, 77 in the intervention group and 64 in the control group. Based on the PSS-14, 70.1% of the intervention group and 75% of the control group (*p* = 0.342) had symptoms of stress. Having contact with COVID-19 patients [OR 4.16(1.64–10.52)]; having minors in charge [OR 2.75 (1.15–6.53)]; working as a doctor [0.39(0.16–0.95)], and being a woman [OR 2.94(1.09–7.91)] were related with PSS14 symptoms. Based on the HADS, 54.6% of the intervention group and 42.2% of the control group (*p* = 0.346) had symptoms of anxiety or depression. Having contact with COVID-19 patients [OR 2.17(1.05–4.48)] and having minors in charge [OR 2.14(1.06–4.32)] were related to HADS symptoms. Healthcare professionals who attended COVID-19 patients showed higher levels of anxiety and depression [OR 2.56(1.03–6.36) (*p* = 0.043)].

**Conclusion:**

Healthcare professionals trained in non-technical skills through simulation tended towards higher levels of anxiety and depression and fewer levels of stress, during the COVID-19 pandemic.

## Background

On March 11, 2020, the World Health Organization declared the severe acute respiratory syndrome coronavirus 2 (SARS-CoV-2) outbreak a pandemic disease [1]. Emotionally traumatic experiences such as this pandemic can cause stress-related disorders [2, 3]. Indeed, we previously reported that 65% of a Spanish sample of 5000 individuals reported anxiety or depressive symptoms during the COVID-19 outbreak [4].

Healthcare professionals (HP) are a high-risk population group since they have faced a huge challenge and psychological stress, anxiety, and depression from pandemics [5, 6]. Indeed, given the critical situation that we are experiencing, HP who are directly involved in the diagnosis, treatment, and care of COVID-19 patients may develop symptoms related to psychological suffering that can trigger other mental health symptoms.

The unique nature of intense working relationships in crises requires not only technical skills but also non-technical competencies related mainly to leadership, communication, and situation awareness [7]. To prepare people for these scenarios, simulation-based teamwork training (SBTT) becomes an essential resource, since it should help to develop the skill and competency of HP to cope with the stress associated with emergencies and disasters [8]. Contexts of education and professional practice have profound effects on the substance and quality of learning outcomes and on how professional competence is expressed clinically [9]. SBTT offers learning in a safe container with a high-fidelity psychological environment, without repercussion on the patient. In addition to training non-technical skills, the acute stress responses in SBTT do not differ when comparing with real situations [10]. Besides, it has been observed that in simulation scenarios, biological stress markers are detected [11] and that the performance of the team in stressful situations is a function of the non-technical skills of the leader [12]. This suggests that simulation can be a powerful ally to prepare HP for better coping in stressful situations, even showing a decrease in work stress levels in nurses from intensive care units, keeping the positive effects at 6 months of follow-up [13].

COVID-19 has become the biggest health crisis in decades and has become a stressful context for HP. There is a lack of knowledge about the impact of an SBTT intervention on stress, anxiety, and depression levels in this health emergency. This work aims to study the effect of an SBTT program on stress, anxiety, and depression levels during the onset of the COVID-19 crisis in HP.

## Methods

### Study design

This was a prospective observational cohort study to determine the effect of having received an SBTT on stress, anxiety, and depression levels. The study was carried out in a single tertiary hospital. With the declaration of COVID-19 as a pandemic, our hospital became a COVID-19 reference centre, admitting more than 2700 patients during the first two months.

### Participants

All HP who had received an SBTT, 48 doctors and 48 nurses from different medical specialties, were invited to participate in a psychological survey and were designated as the intervention group (IG). The control group (CG) was built by inviting to participate 134 HP who had no contact with simulation training.

### Intervention courses

The Vall Hebron Advanced Clinical Simulation centre (VHiSCA), has conducted three editions (from November 2019 to March 2020), of an SBTT instructor course, 32 HP having participated in each one. The course had a mixed design with a total of 25 h and was divided into online (13 h) and on-site practical part (12 h). As VHISCA has just started the formation with HP we had implemented it with medical practitioners and not with delegates yet. The main objectives were to provide basic knowledge on healthcare simulation, to provide skills to be a simulation instructor, to acquire non-technical skills management, and to master briefing and debriefing. From our SBTT instructor course database we know that only 9% of the participants had taken a simulation instructor course prior to the intervention.

### Stress, anxiety and depression instrument

A Survey format was designed including two scales. The Hospital Anxiety and Depression Scale (HADS) [14, 15] was applied to assess the level of anxiety and depression. The HADS has been widely used in the clinical setting and has been validated for the Spanish population [15] and demonstrated an optimal internal consistency and test-retest reliability in the Spanish non-clinical population [16]. It is composed of 14 items and evaluates 7 items for each dimension. In this study, scores > 12 were considered positive for anxiety and depression domain [15]. A 12–15 cut-off could be appropriate for a non-clinical population [16] with a sensibility and specificity of over 70% [17]. The Perceived Stress Scale-14 (PSS-14) [18, 19] was used to evaluate stress. This scale has 14 items that evaluate stress perceived in the last month. Both scales have already been used in studies with HP [20, 21] and responded with a four-point Likert scale. Based on previous literature [16, 18, 19] we analysed groups with cut-off scores, HADS> 12 and PSS14 > 18.

### Data collection and ethical approval

After approval by the ethical committee of Vall d’Hebron University Hospital (PR (AG)261/2020), and within the third month of the pandemic**,** HP were invited to participate in the study through corporate email. Informed consent was asked, and data was obtained anonymously. The survey was first sent to the IG. To collect data for the CG, participants were asked to invite a co-worker with no experience in simulation to participate.

### Data analysis

For the descriptive analysis, categorical data are shown as frequency and percentage, and differences between groups were analysed using the chi-square test. For continuous variables, normal distribution was tested by the Kolmogorov-Smirnov test; data following a normal distribution is shown as mean and standard deviation and differences were analysed using the Student’s t-test.

Effect sizes for continuous variables were analysed using the between-group mean difference (95% confidence interval). Logistic regression was used to assess whether having attended the simulation-based teamwork training program modified the probability of having stress, anxiety, or depressive symptoms. The effects of sex, age, working with COVID-19 positive patients, having minors in charge, being married and professional category were considered. Sub-analyses were performed based on exposure to COVID-19. The statistical significance level was set at *p* < 0.05. Statistical software packages STATA 15.1 was used for all data analyses.

## Results

A total of 141 HP completed the online survey, 77 (80.2%) in the IG, and 64 (47.8%) in the CG. The demographic characteristics of both groups are shown in Table 1.

**Table 1:** Baseline demographic characteristics. Data were expressed as frequency (%) and mean (SD)

	Instructors (*n* = 77)	Controls (*n* = 64)	*p*
Sex			
Female	54 (70.1%)	54 (84.4%)	0.047
Male	23 (29.9%)	10 (15.6%)	
Age (years)	41.9 (8.1)	38.5 (9.1)	0.020
Couple / married	39 (50.6%)	26 (40.6%)	0.234
Minors	45 (58.4%)	26 (40.6%)	0.035
Professional category			0.633
Doctors	38 (49.4%)	29 (45.3%)	
Nurses	39 (50.6%)	35 (54.7%)	
Workplace: COVID area	51 (66.2%)	37 (57.8%)	0.304
Contact with COVID-19 patients			0.282
Daily	42 (54.6%)	34 (53.1%)	
Weekly (> 1 day per week)	28 (36.4%)	19 (29.7%)	
Occasionally (< 5 days per month)	4 (5.2%)	3 (4.7%)	
Rarely	3 (3.9%)	8 (12.5%)	

In the IG, the HADS score mean was 14.23 (SD 7.41) and in the CG was 12.08 (SD 6.66), mean difference − 2.15 (95% CI -4.52 – 0.22). Based on the HADS, 54.6% of the IG and 42.2% of CG (*p* = 0.346) had symptoms of anxiety or depression. Significant differences were observed only in HP in contact with COVID-19 patients (*p* = 0.037) and having minors in charge (*p* = 0.033) (Table 2).

**Table 2:** Logistic regression of variables related with HADS > 12 and PSS14 > 18 between intervention and control group

		Odds Ratio (95% CI)	***p***
**HADS > 12**	COVID-19	2.17 (1.05–4.48)	0.037
	Minors in charge	2.14 (1.06–4.32)	0.033
**PSS14 > 18**	COVID-19	4.16 (1.64–10.52)	0.003
	Minors in charge	2.75 (1.15–6.53)	0.022
	Working as doctor	0.39 (0.16–0.95)	0.038
	Women	2.94 (1.09–7.91)	0.033

PSS-14 score mean was 23.92 (SD 8.65) in the IG and 24.33 (SD 9.00) in the CG, mean difference 0.41 (95% CI -2.54 – 3.36). Based on the PSS-14, 70.1% of the IG and 75% of the CG (*p* = 0.342) had symptoms of stress. Significant differences were observed in HF in contact with COVID-19 patients (*p* = 0.003), having minors in charge (*p* = 0.022), working as a doctor (*p* = 0.038), and being a woman (*p* = 0.033) (Table 2).

Based on previous results, we performed a sub-analysis only with HP who had attended COVID-19 patients (*n* = 88), 51 (58%) in the IG, and 37 (42%) in the CG Healthcare professionals that had attended COVID-19 patients showed higher levels of anxiety and depression [OR 2.56 (1.03–6.36) (*p* = 0.043)]. (Table 3).

**Table 3:** Logistic regression of variables related to HADS> 12 and PSS14 > 18 between intervention and control group depending on the hospital area where they worked (COVID or No COVID). *SBTT* simulation-based teamwork training

		Odds Ratio (95% CI)	***p***
**COVID-19**			
HADS> 12	Performance SBTT	2.56 (1.03–6.36)	0.043
	Working as doctor	0.35 (0.132–0.94)	0.038
PSS14 > 18	Working as doctor	0.19 (0.06–0.60)	0.005
**No COVID-19**			
HADS> 12	Minors in charge	4.25 (1.17–15.45)	0.028
PSS14 > 18	Minors in charge	4.14 (1.21–14.12)	0.023
	Sex Female	5.74 (1.21–36.26)	0.63

## Discussion

This study provides results about the impact of an SBTT program on stress, anxiety, and depression in HP during a crisis. We found that in the IG when they were exposed to COVID-19 care patients, they suffered more anxiety and depression levels (HADS) than the HP who did not receive the education SBTT program. To our knowledge, this is the first study focusing on mental health symptomatology on SBTT participants.

HP taking care of patients with COVID-19 are at risk of developing psychological distress and other mental health symptoms [22]. Numerous psychiatric or psychological departments, psychological counselling centres, and university psychology units have launched specialized programs to attend HP in hospitals, through telematics systems, telephone lines, and apps, to provide psychological treatment in crisis. In this context, we found that HP who have received an SBTT program presented more anxiety and depression when dealing with COVID-19 patients. Firstly, higher perception and awareness levels are observed after SBTT [23], and usually, these professionals are more involved and committed in education tasks in hospitals, entailing otherwise more responsibility tasks. Another study found similar results showing a relationship between awareness attitude, anxiety experience, and perceived mental health care needs in the community during the COVID19 pandemic [24]. Secondly, collective efficacy was independently associated with flow in simulation practice, but no significant results were found for stress and self-esteem [25].

As in our study conducted in the general population [4], we observed that individuals with minors in charge and women had higher anxiety and depressive symptoms. Besides, we also found that HP who worked with COVID-19 patients had more symptoms of stress, anxiety, and depression, and women had more symptoms of stress probably related to the working environment, huge workload, long-term fatigue, infection threat, and frustration with the death of patients whom they care. There is evidence that healthcare simulation improves healthcare performance and degree of retention of what is learned when compared to all traditional teaching methods [26]. Our study results show that the emergency together with the knowledge of these abilities by part of the entire working group may be determining factors for the psychological state of the skills previously trained by using SBTT.

Despite that, it is necessary to keep working on stress levels in HP during the simulation and in real settings. Simulation educators need to create scenarios that purposely change stress to enhance learning as it was found that psychological stress and anxiety is greater during simulation compared with hospital settings [27]. The intervention used is a faculty training programme rather than a simulation based education programme for delegates. Subsequent research should focus on studying the degree of distress that students present in crisis situations based on the profile of the educator. Besides, health professions education programs must build student competencies in the affective domain of learning [28].

For this reason, it is crucial to be able to train professionals, promoting skills through SBTT that increases job security and reduces levels of distress generated in crises, both in technical aspects (performing cardiopulmonary resuscitation, placement of line or tube, tracheostomy, etc.) as in non-technical skills (communication, situational awareness, etc.) because stress triggers diseases and increases health resources.

### Strengths and limitations

Study strengths are that it is the first study focus on stress, anxiety, and depression in simulation instructors after a pandemic, and it combined different new areas never studied before, such as professional’s health and simulation training after COVID-19.

We acknowledge that our survey has several limitations. Firstly, there is likely to have been a degree of selection bias in the CG. Secondly, the number surveyed varied greatly between the two groups. Thirdly, we cannot rule out the presence of residual confounders.

## Conclusion

HP trained in non-technical skills as simulation instructor had a tendency towards higher levels of anxiety and depression and fewer levels of stress, during the COVID-19 pandemic, being significant in the case of anxiety and depression in HP who directly attended COVID-19 patients. Studies with structured interventions, randomized controlled trials, with larger homogeneous samples, can provide more information. Therefore, more studies with these characteristics should be done before being able to conclude the effectiveness of simulation-based techniques.

## Data Availability

The datasets used and/or analyzed the current study are available from the corresponding author on reasonable request.
